# Free Riding in Healthcare Through a Game-Theoretic Lens: A Cross-Domain Narrative Review and Conceptual Synthesis

**DOI:** 10.3390/healthcare14121651

**Published:** 2026-06-11

**Authors:** Christos Ntais, Michael A. Talias

**Affiliations:** Healthcare Management Program, School of Economics & Management, Open University of Cyprus, Nicosia 2220, Cyprus; christos.ntais@st.ouc.ac.cy

**Keywords:** free riding, game theory, public goods, health insurance, vaccination, antimicrobial resistance, organ donation, global health, health policy

## Abstract

**Background/Objectives:** Free riding in healthcare occurs when actors benefit from health-related public goods, risk-pooling arrangements, common resources, or cooperative institutions while contributing less than is socially optimal. This review clarifies how free-rider dynamics differ across vaccination, health insurance and universal health coverage, antimicrobial resistance, organ donation and transplant allocation, and global health cooperation. **Methods:** A narrative review with conceptual synthesis was conducted. Searches of PubMed and Scopus were complemented by citation tracking and targeted inclusion of foundational economics, game theory, public-health ethics, and market-design sources. Sources were mapped by domain, actors, strategies, payoff structure, information conditions, time horizon, enforcement mechanism and policy relevance. **Results:** Across domains, free riding arises when private payoffs diverge from collective welfare, but the underlying game differs: threshold public-good and coordination games in vaccination, adverse-selection and participation games in insurance, common-pool-resource dilemmas in antimicrobial use, donor-registration and matching-market problems in transplantation, and repeated public-goods games in global health. The review identifies three policy functions: altering payoffs, altering information and beliefs, and changing the structure, repetition, or enforceability of the game. **Conclusions:** Game theory is most useful as a mechanism-based framework rather than a stand-alone policy prescription. Its policy value depends on empirical calibration, institutional context, ethical legitimacy, and attention to equity, incomplete information, behavioral responses, and enforcement capacity. The synthesis also emphasizes boundary conditions: game-theoretic prescriptions can fail when political economy, asymmetric power, implementation capacity, access barriers, or trust-related drivers are ignored.

## 1. Introduction

The free-rider problem is a collective-action problem in which actors benefit from a good, resource, or institutional arrangement without contributing at a level that would be required for its efficient and equitable provision [1,2]. In healthcare, the term should not be applied indiscriminately to all care. Some health goods are close to public goods, such as disease surveillance and herd immunity; some are common-pool resources, such as antimicrobial effectiveness; some are club or risk-pooling arrangements, such as insurance pools; and many clinical services are private or rival goods delivered within publicly or privately financed systems. Distinguishing these categories is essential because the relevant game, strategic actors and policy instruments differ across contexts [3].

Game theory is useful because it focuses on strategic interdependence: the payoff from an individual, provider, insurer, government, or state decision depends partly on what others do [4,5]. Behavioral economics and public-health ethics are complementary rather than competing frameworks. Behavioral economics helps explain why actors do not always maximize expected utility; public-health ethics evaluates whether incentives, mandates, surveillance, or sanctions are legitimate. Game theory adds a mechanism-based language for specifying players, strategies, payoffs, information, repetition, enforcement and equilibrium outcomes.

The comparative claim of this review is therefore deliberately modest. Game theory is not treated as superior to behavioral economics, sociology of cooperation, institutional analysis, or public-health ethics in all circumstances. Its distinctive contribution is strongest when strategic interdependence, externalities, incentives, information asymmetry, repeated interaction, or enforcement explain under-contribution. It is weaker when non-participation is primarily caused by access barriers, mistrust, misinformation, coercion, or structural inequity; in those settings, game-theoretic models should be combined with behavioral, institutional and ethical analysis rather than used alone.

Prior work has examined game-theoretic or free-rider dynamics within individual domains, especially vaccination [6,7,8,9], antimicrobial prescribing [10], health insurance [11,12,13,14,15], organ donation [16,17,18,19] and global vaccine cooperation [20]. However, these studies are often siloed. The analytical gap addressed here is not the absence of domain-specific models, but the lack of a cross-domain synthesis that compares how free riding changes when the underlying strategic problem shifts from a threshold public good to adverse selection, common-pool-resource depletion, donor-registration participation, market design, or repeated international cooperation.

The scope of this review is therefore deliberately bounded. Five healthcare domains in which individual or institutional choices generate externalities for others were examined: vaccination and herd immunity, health insurance and universal health coverage, antimicrobial resistance, organ donation and transplant allocation and global health initiatives. Purely clinical allocation decisions that do not involve strategic contribution to a shared good were excluded, except where they intersect with organ donation or market-design mechanisms.

The aim is to develop an integrated conceptual framework that (i) identifies the strategic actors and payoff structures associated with free riding in each domain, (ii) clarifies the relevant game-theoretic mechanisms and equilibrium problems, (iii) links policy interventions to their game-theoretic function, and (iv) specifies the limitations of translating stylized models into healthcare policy.

## 2. Materials and Methods

This article is a narrative review with structured search, transparent source mapping, and conceptual synthesis. The structured elements improve reproducibility and reduce selective interpretation while preserving the interpretive purpose of a narrative conceptual review [21,22].

Searches were conducted in PubMed and Scopus for publications available up to and including May 2026. Search strings combined game-theoretic and collective-action terms with healthcare domains: “game theory”, “game theoretic”, “free rider”, “free rid*”, “public goods”, “collective action”, “healthcare”, “health care”, “health system”, “health policy”, “vaccination”, “health insurance”, “universal health coverage”, “antimicrobial resistance”, “organ donation”, “transplant allocation”, and “global health”. Since PubMed and Scopus may underrepresent economics and political–economy sources, backward and forward citation tracking and targeted inclusion of foundational works in insurance economics, common-pool-resource theory, public-health ethics, market design and antimicrobial-resistance policy were employed [3,13,14,15,19,21,22,23,24,25,26].

Eligible sources were English-language peer-reviewed empirical studies, theoretical papers, reviews, policy analyses and academic book chapters that addressed at least one of the following: free riding, collective action, public-good provision, adverse selection, common-pool-resource depletion, strategic contribution, game theory, market design, incentive compatibility, or enforcement in healthcare or public health. Sources were excluded if they mentioned game theory or free riding only tangentially, addressed healthcare without strategic interdependence, lacked relevance to incentive design or institutional cooperation, or were not available in English.

A formal risk-of-bias assessment was not undertaken because the included sources were heterogeneous in design and purpose, including mathematical models, ethical analyses, policy papers, empirical studies and narrative or systematic reviews. This limits the ability to rank causal certainty across interventions. To make this limitation explicit, the synthesis distinguishes between conceptual mechanisms, empirical examples and policy implications. Policy recommendations are therefore framed as mechanism-informed options requiring context-specific empirical evaluation rather than as definitive estimates of effectiveness.

To reduce the risk that a narrative synthesis could overstate positive applications, the extraction also recorded cautionary and negative implications when identified, including weak transferability of stylized models, crowding out of intrinsic motivation, inequitable burdens, enforcement problems, privacy risks, and potential strategic manipulation. These observations were not treated as effect estimates, but as boundary conditions for interpreting policy relevance.

## 3. Source Mapping and Integrated Analytical Framework

**Figure 1:**
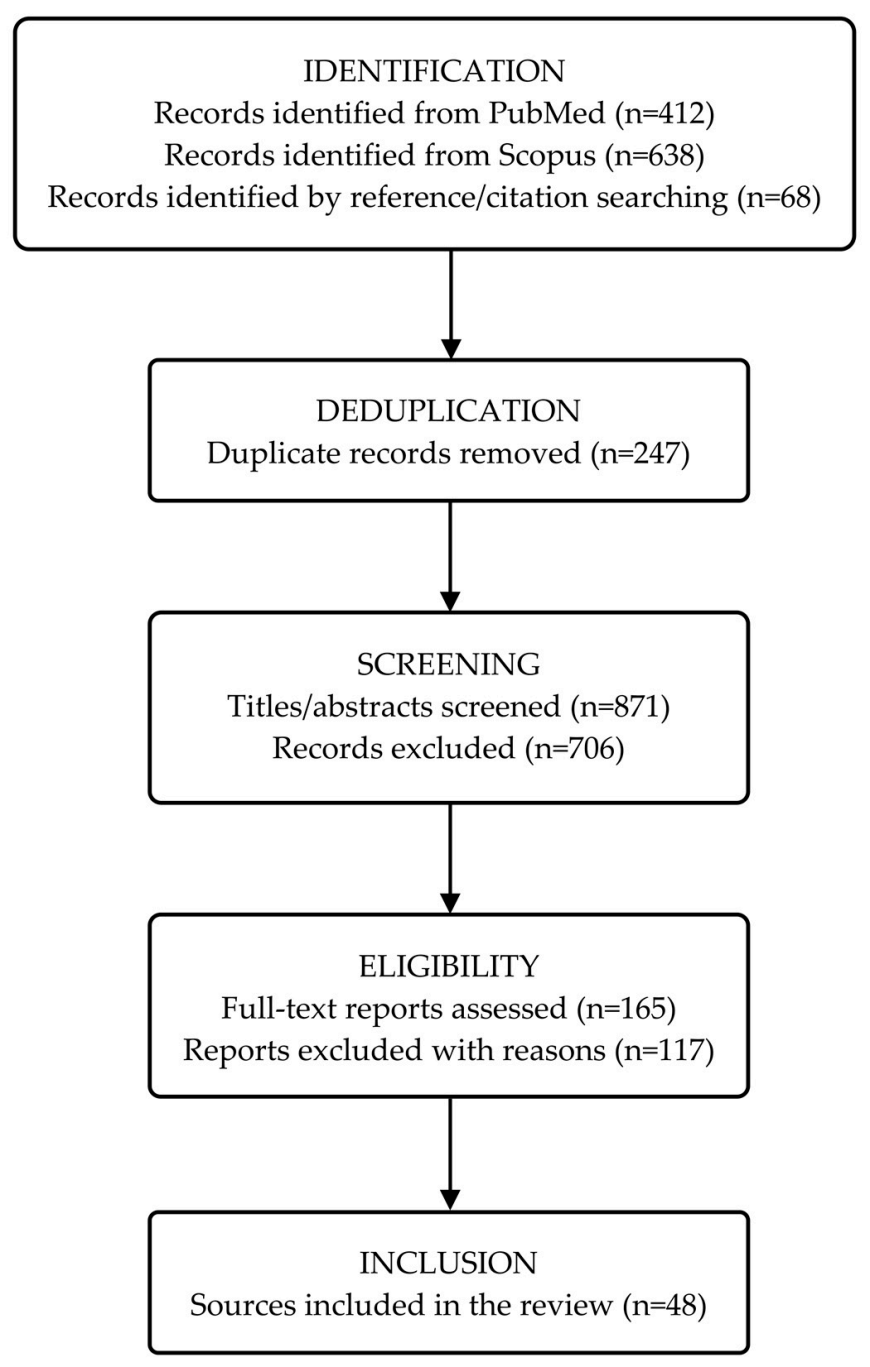
Selection-flow diagram for the structured narrative review.

The search and selection pathway is summarized in Figure 1. In total, 1118 records were identified (412 from PubMed, 638 from Scopus, and 68 from reference/citation searching). After removal of 247 duplicates, 871 titles and abstracts were screened and 706 records were excluded. A total of 165 full-text reports were assessed; 117 were excluded because they were outside healthcare/public health (n = 31), mentioned game theory or free riding only tangentially (n = 39), lacked a strategic or incentive-design contribution (n = 23), or were unavailable, non-English, or duplicative reports (n = 24). Forty-eight sources were retained for the conceptual synthesis.

Table 1 summarizes the evidence map used for conceptual synthesis, while Table 2 presents the cross-domain analytical framework.

The synthesis showed that the same label—free riding—covers different strategic mechanisms. In vaccination, individuals may rely on others to sustain herd immunity; in insurance, low-risk individuals may opt out of risk pools when premiums exceed perceived private benefit; in antimicrobial use, immediate private treatment gains can erode the shared future effectiveness of antibiotics; in organ donation, free riding is most applicable to donor registration or willingness to contribute organs, whereas transplant allocation itself is primarily a matching and market-design problem; and in global health, states may underinvest in surveillance, preparedness, or equitable countermeasure distribution while benefiting from the efforts of others.

Across the five domains, policy interventions worked through three broad game-theoretic functions: altering payoffs, altering information and beliefs, and changing the structure or repetition of the game. These functions provide a more integrated framework than listing interventions by domain alone.

## 4. Domain-Specific Game-Theoretic Analysis

### 4.1. Vaccination Uptake

Vaccination decisions are often modeled as strategic choices under externalities. Let c_i_ denote the perceived private cost of vaccination for individual i, L_i_ the expected loss from infection, q(x) the infection risk when vaccination coverage in a population of size N is x, and x_−i_ the vaccination coverage excluding person i. Individual i vaccinates when the private expected benefit, [q(x_−i_) − q(x_−i_ + 1/N)]L_i_ plus any perceived altruistic or social benefit, exceeds c_i_. The social planner, however, also counts the reduced infection risk imposed on others. Free riding appears when c_i_ exceeds the private benefit but is lower than the private plus external benefit.

The equilibrium concern is that voluntary uptake may stabilize below the socially optimal threshold if individuals discount external benefits or believe that others will vaccinate. Depending on disease transmissibility, perceived risk, vaccine effectiveness and social norms, the relevant game can resemble a threshold public-good game, a coordination game, or a “snowdrift”/volunteer’s dilemma rather than a single universal prisoner’s dilemma [6,7,8,9,27].

Empirical and experimental studies support the relevance of free-riding motives but also show that behavior is shaped by trust, perceived risk, social norms, mandates, misinformation and institutional context [8,9,28,29,30,31,32]. Therefore, game-theoretic models should be calibrated with empirical data rather than interpreted as literal descriptions of all vaccination decisions.

### 4.2. Health Insurance and Universal Healthcare Coverage

In insurance markets, the free-rider problem overlaps with adverse selection and risk pooling. Let P be the premium or contribution, E_i_ the individual’s expected healthcare cost, R_i_ the value of risk protection and M the mandate penalty or loss of access associated with non-participation. A low-risk individual may opt out when P > E_i_ + R_i_ + M. If many lower-risk individuals leave the pool, average cost rises, premiums increase and the pool may become progressively more adverse selected [11,12,13,14,15].

This is not a pure public-good game because insurance is an excludable contract or entitlement. It is a strategic participation game in which private information about risk, affordability, subsidies and expected use affects enrollment. Universal coverage, individual mandates, premium subsidies, automatic enrollment and risk adjustment can alter the payoff structure by reducing the advantage of opting out and stabilizing the pool.

Empirical work on mandates and adverse selection shows that real-world effects depend on enforcement, subsidy design, premium levels and population risk distribution [14,15]. Thus, the policy implication is not simply that mandates are always optimal, but that risk-pool participation requires credible rules and affordability mechanisms that align individual and collective incentives.

### 4.3. Antimicrobial Resistance

Antimicrobial effectiveness is a common-pool resource. Patients, prescribers, hospitals, agriculture and governments may gain immediate private benefit from antibiotic use while imposing diffuse future costs through resistance [10,23,24,25]. Let b_i_ be the private benefit from antibiotic use, c_i_ the private cost and Σ_j_E_j_ the total external resistance costs imposed on others. A simplified condition for overuse is b_i_ − c_i_ > 0 for the individual prescriber or patient, while b_i_ − c_i_ − Σ_j_E_j_ < 0 from a social perspective.

The Nash-equilibrium tendency in an unregulated, one-shot setting is overuse relative to the social optimum because each actor internalizes only a small share of the future resistance cost. Repeated interaction, institutional monitoring, prescribing feedback, audit-and-feedback systems, formularies, diagnostic stewardship and penalties for inappropriate prescribing can change the game from one-shot extraction to governed stewardship.

The policy relevance of this model depends on recognizing incomplete information. Prescribers often face diagnostic uncertainty and medico-legal pressure, while patients may expect antibiotics. Stewardship policies should therefore combine payoff changes with better diagnostics, clinical decision support and trust-building rather than relying solely on sanctions.

### 4.4. Organ Donation and Transplant Allocation

Organ donation requires conceptual separation between two strategic problems. Donor registration and willingness to donate can involve free riding: individuals may support the existence of transplant systems and expect access as recipients while not registering as donors or encouraging donation. In contrast, allocation of organs among recipients is primarily a matching, prioritization and market-design problem rather than a classical free-rider problem [16,18,33].

The strategic actors differ by stage. In donor registration, actors are potential donors, families, registries and policymakers; strategies include registering, not registering, consenting, refusing, or accepting default options. In allocation, actors include transplant centers, allocation agencies, recipients and donor–recipient pairs; strategies concern listing, reporting, matching, exchange participation and acceptance of offers. Matching algorithms such as kidney exchange improve efficiency and incentive compatibility, but they do not by themselves solve donor-supply free riding [34,35].

Default-choice evidence, including opt-in versus opt-out systems, shows that framing and transaction costs influence donor registration [19]. Therefore, this section now uses the term “free riding” only for the participation and contribution dimension of organ donation and treats transplant allocation as a related but distinct game-theoretic market-design problem.

### 4.5. Global Health Initiatives

Global health cooperation often takes the form of repeated public-goods games among states, international organizations, funders, manufacturers and civil-society actors. Pandemic preparedness, disease surveillance, antimicrobial-resistance surveillance, vaccine research and equitable countermeasure distribution generate cross-border benefits. Individual states may underinvest if they expect others to finance the infrastructure or may hoard countermeasures when domestic political payoffs dominate global welfare [20,36].

The equilibrium concern is under-provision of global public goods and inefficient allocation during crises. Unlike stylized symmetric games, global health games involve unequal power, fiscal capacity, manufacturing control and vulnerability. These asymmetries affect bargaining, enforcement and the fairness of contribution rules.

Repeated interaction, reputational costs, conditional financing, treaty commitments, pooled procurement, advance-purchase commitments and side payments can improve cooperation. However, such mechanisms are credible only when they incorporate monitoring, transparency and perceived fairness.

Table 3 summarizes the formal free-riding conditions and equilibrium implications by domain discussed in this review.

## 5. Discussion

The cross-domain comparison suggests that game theory should be used less as a catalog of named games and more as a diagnostic method. The first question is not “Which famous game applies?” but “Who are the actors, what do they know, what do they choose, what do they gain or lose, how often do they interact and how are agreements enforced?” This approach prevents overextension of the free-rider label and clarifies why a policy suitable for vaccination may not be suitable for organ allocation or antimicrobial prescribing.

Policy mechanisms that alter payoffs include subsidies, penalties, mandates, insurance contributions, premium subsidies, stewardship restrictions, rewards for donor registration and conditional international financing. Their purpose is to reduce the private advantage of non-cooperation or increase the private return from cooperation. These mechanisms require careful calibration because poorly designed incentives can crowd out intrinsic motivation, burden disadvantaged groups, or trigger public resistance [37,38].

Policy mechanisms that alter information and beliefs include transparent risk communication, social-norm messaging, audit-and-feedback, public dashboards, decision aids, diagnostic information and digital reminders. These interventions affect perceived probabilities, perceived costs and expectations about others’ behavior. They are especially relevant when non-cooperation is driven by misinformation, distrust, or underestimation of externalities rather than by deliberate opportunism [9,31,32,39,40,41].

Policy mechanisms that change the structure or repetition of the game include automatic enrollment, opt-out defaults, repeated institutional reporting, treaty commitments, registries, reputational mechanisms and cooperative organizations. These mechanisms can transform a one-shot temptation to defect into a repeated relationship with monitoring, reciprocity and enforcement [19,20,42].

Ethically, interventions against free riding must satisfy more than efficiency. Public-health ethics emphasizes effectiveness, proportionality, necessity, least infringement, public justification and fair distribution of burdens and benefits [26,43,44]. Coercive interventions may be justified in high-risk settings, but they require transparent reasoning, due process, accessibility of the cooperative option and safeguards against disproportionate effects on marginalized populations.

Digital technologies and artificial intelligence can support the game-theoretic functions above when used with governance. Predictive analytics can identify undervaccinated communities or groups with high hesitancy, natural-language-processing tools can monitor vaccine sentiment and machine-learning decision support can help antimicrobial stewardship by predicting resistance or recommending guideline-concordant therapy [45,46,47]. In game-theoretic terms, these tools may improve information, reduce uncertainty, target interventions and strengthen monitoring. However, they can also introduce privacy risks, algorithmic bias, unequal access, surveillance concerns and erosion of trust [48]. Their use should therefore be evaluated not only for predictive performance but also for fairness, transparency, accountability and acceptability.

A practical implication is that policy levers should be used selectively rather than as a generic menu. Subsidies are most appropriate when socially desirable cooperation is privately costly or access barriers are substantial; they are less appropriate when the main barriers are mistrust or misinformation, when payments risk signaling that the behavior is unusually dangerous, or when benefits are captured by actors who would have cooperated anyway. Penalties and mandates are most defensible when external harms are high, the cooperative option is accessible, and exemptions or due process protect those with legitimate reasons for non-participation. Otherwise, they can harden resistance, shift burdens to disadvantaged groups or crowd out intrinsic motivation [37,38,43,44].

Feasibility and political economy should also be modeled as part of the game. Policymakers, regulators, insurers, professional associations, pharmaceutical firms, hospitals, donors and states are themselves strategic actors. A technically attractive intervention may fail if powerful actors can lobby for exemptions, shift costs to weaker participants, underfund enforcement, or free ride on monitoring performed by others. Asking who pays, who enforces, who can evade, who benefits and who bears risk helps move the analysis from benevolent-planner recommendations to implementable institutional design [3,20,26].

Heterogeneity changes both equilibrium predictions and ethical conclusions. Actors differ in income, risk, health status, contraindications, access, trust, information, bargaining power and market position. A patient who cannot access a vaccination site, afford insurance contributions or comply with a digital monitoring requirement should not be modeled as equivalent to a well-resourced actor strategically avoiding contribution. Conversely, powerful organizations or states may impose larger externalities than individuals. Future models should therefore incorporate asymmetric players, distributional weights, equity constraints or stratified payoff structures rather than relying only on representative-agent assumptions [26,43,44].

Several cautionary cases illustrate when game theory can mislead. A model that treats vaccine refusal solely as strategic free riding may recommend sanctions while missing trust-building and access problems. An antimicrobial-resistance model that treats every prescription as opportunistic overuse may ignore diagnostic uncertainty and medico-legal pressure. A donor-default model may raise supply but also trigger legitimacy concerns if citizens perceive consent as manipulated. A digital-monitoring model may increase observability while undermining trust through surveillance, bias or unequal access [10,19,43,48].

Table 4 summarizes mitigation strategies by game-theoretic function discussed in this review.

## 6. Limitations

This review has several limitations. First, it is a narrative conceptual synthesis rather than a systematic review, scoping review, or meta-analysis. The search and selection process was structured, but the included corpus remains interpretive and may omit relevant sources, especially from economics, political science, sociology, ethics, or law, that are not indexed in PubMed and may be imperfectly captured by Scopus.

Second, no formal risk-of-bias assessment was conducted because the evidence base included heterogeneous source types: mathematical models, empirical studies, reviews, policy analyses and ethical arguments. This limits the credibility of any causal claim that a particular intervention will work across settings. Accordingly, the conclusions should be read as mechanism-informed hypotheses and policy design considerations rather than as pooled estimates of effectiveness.

Third, game-theoretic models simplify healthcare behavior. Real actors operate under incomplete information, bounded rationality, social norms, institutional constraints, inequitable resource distribution and political pressures. Equilibrium predictions can change when payoffs are dynamic, actors learn, trust changes, or enforcement is weak.

Fourth, the free-rider concept has limits. It is analytically useful when actors benefit from a cooperative good while under-contributing, but it is misleading when applied to all allocation problems or to people whose non-participation reflects poverty, access barriers, medical contraindications, historical mistrust, or coercive policy environments.

Fifth, although the review discusses implementation barriers and failure modes, it does not provide a formal political–economy model or a comparative empirical case-study analysis of successful versus failed interventions. The cross-domain synthesis should therefore be read as a framework for identifying where empirical calibration, asymmetric-player modeling and implementation research are needed, not as proof that game theory outperforms alternative frameworks.

## 7. Future Directions

Future research should develop empirically calibrated models rather than relying on illustrative payoff matrices. In vaccination, this requires linking perceived risk, trust, social norms and local coverage thresholds. In insurance, it requires estimating how subsidies, penalties, enrollment defaults and risk adjustment affect participation across income and risk groups. In antimicrobial stewardship, it requires models that include diagnostic uncertainty and institutional prescribing norms.

Future work should also combine game theory with behavioral economics and ethics. Behavioral models can explain why actors may cooperate despite short-term incentives to defect and ethical analysis can determine when mandates, sanctions, surveillance, or algorithmic targeting are legitimate.

Digital and AI-enabled systems should be evaluated as interventions that alter information, monitoring and enforcement in strategic environments. Research should test whether predictive tools improve cooperation without worsening inequities, undermining privacy, or reducing trust. Transparent reporting, bias audits, community engagement and prospective evaluation should accompany any digital free-rider mitigation strategy.

A next generation of studies should compare cases in which game-theoretic interventions improved cooperation with cases in which they failed, generated perverse incentives, or were politically infeasible. Such work should model asymmetric actors and second-order free riding among regulators, payers, manufacturers, professional groups and states, and should test when behavioral, sociological or institutional frameworks explain more than strategic payoff models.

## 8. Conclusions

Free riding in healthcare is not a single problem with a single solution. It appears in different forms across vaccination, health insurance, antimicrobial resistance, organ donation and global health cooperation. Game theory helps by clarifying the strategic structure behind each problem: who benefits, who contributes, who bears external costs, what information is available, whether interactions repeat and how cooperation is enforced.

The most robust cross-domain insight is that policies should be matched to the game they seek to change. Payoff-changing mechanisms, information-changing mechanisms and structure-changing mechanisms each have a role, but their ethical acceptability and effectiveness depend on empirical context, equity, legitimacy and institutional capacity. Used carefully, game theory can help health policymakers design more coherent interventions; used simplistically, it risks reducing complex social and ethical problems to misleading labels.

The practical contribution of game theory is therefore conditional rather than universal: it is most valuable when it exposes a specific strategic mechanism that can be empirically tested, ethically justified and institutionally enforced. It is least useful when it turns access barriers, distrust, coercion or structural injustice into a simplified accusation of free riding.

## Figures and Tables

**Table 1:** Evidence map and analytical role of included source types.

Domain	Representative Source Types	Key Analytical Contribution	Limitations Noted
Vaccination	Game-theoretic models, experiments, behavioral and social-norm studies	Threshold public-good dynamics; private versus social vaccination benefits; empirical role of trust and norms	Payoffs vary by disease, perceived risk, vaccine access, contraindications and misinformation
Insurance/UHC ^1^	Health economics, adverse-selection theory, mandate and risk-pool literature	Strategic participation and risk-pool stability; opt-out incentives among lower-risk individuals	Not a pure public good; affordability and institutional design are central
AMR ^2^	Common-pool-resource theory, prescribing models, stewardship policy	Private short-term treatment benefit versus diffuse future resistance cost	Diagnostic uncertainty and provider constraints complicate simple overuse models
Organ donation	Ethics, donor registration, defaults, matching markets, kidney exchange	Free riding applies mainly to donor participation; allocation is a market-design problem	Donor supply, family consent and allocation efficiency are distinct mechanisms
Global health	Collective-action and repeated-game analyses, pandemic cooperation literature	Underinvestment and inequitable distribution in cross-border public goods	Power asymmetries and weak enforcement limit simple cooperation models

^1^ UHC: Universal Health Coverage; ^2^ AMR: Antimicrobial Resistance.

**Table 2:** Cross-domain game-theoretic framework for free riding in healthcare.

Domain	Players and Strategies	Free-Rider Mechanism	Equilibrium/Inefficiency	Policy Levers
Vaccination	Individuals choose vaccinate/not; governments choose communication, mandates, access policies	Non-vaccinated individuals benefit from others’ contribution to herd immunity	Voluntary uptake may fall below social threshold	Reduce cost, increase access, social-norm messaging, mandates in high-risk contexts
Insurance/UHC ^1^	Individuals enroll/opt out; payers and states set premiums, subsidies, penalties	Lower-risk actors may avoid contributions while relying on future access or emergency care	Adverse selection and unstable risk pools	Automatic enrollment, subsidies, individual mandates, risk adjustment
AMR ^2^	Prescribers/patients use antibiotics prudently/liberally; institutions monitor or do not	Immediate private benefit while resistance costs are externalized	Antibiotic overuse and declining effectiveness	Stewardship, diagnostics, audit-feedback, prescribing restrictions
Organ donation	Potential donors register/not; families consent/refuse; centers participate in exchanges	Potential recipients may support access without registering or contributing to donor supply	Insufficient donor pool; matching inefficiency if participation incentives are weak	Defaults, registry design, public trust, exchange algorithms, transparency
Global health	States fund/share/coordinate or underinvest/hoard; organizations monitor and coordinate	States benefit from global preparedness while shifting cost to others	Under-provision of surveillance, preparedness and equitable access	Treaties, pooled procurement, financing rules, reputation, side payments

^1^ UHC: Universal Health Coverage; ^2^ AMR: Antimicrobial Resistance.

**Table 3:** Formal free-riding conditions and equilibrium implications by domain.

Domain	Formal Condition for Free Riding or Under-Cooperation	Equilibrium Implication
Vaccination	Private benefit of vaccination < perceived cost, while private + external benefit > cost	Voluntary coverage below social optimum unless beliefs, costs, or mandates change
Insurance/UHC ^1^	Premium/contribution > perceived expected benefit + risk-protection value + penalty	Lower-risk opt-out and adverse selection unless pooling rules or subsidies change
AMR ^2^	Private benefit of antibiotic use − private cost > 0 while social net benefit < 0 after resistance externality	Overuse in one-shot or weakly monitored settings
Organ donation	Expected benefit from transplant system > private/social motivation to register or consent	Donor supply below socially desired level; matching algorithms address allocation but not supply alone
Global health	National payoff from under-contribution/hoarding > national payoff from cooperative contribution given others’ actions	Underinvestment and inequitable access unless repeated-game and enforcement mechanisms are credible

^1^ UHC: Universal Health Coverage; ^2^ AMR: Antimicrobial Resistance.

**Table 4:** Mitigation strategies organized by game-theoretic function.

Mechanism Category	Game-Theoretic Function	Healthcare Examples	Risks/Cautions
Alter payoffs	Changes the private return from cooperation or defection	Subsidies, penalties, mandates, premium support, stewardship restrictions, donor incentives	Crowding out intrinsic motivation, inequitable burdens, public resistance
Alter information and beliefs	Changes perceived risk, benefits, expectations, or trust	Risk communication, social-norm messages, audit-feedback, diagnostic tools, digital reminders	Misinformation, low trust, unequal access to reliable information
Change structure/repetition/enforcement	Makes cooperation repeated, monitored, defaulted, or institutionally enforceable	Automatic enrollment, opt-out donor defaults, registries, reputation, treaties, pooled procurement	Privacy, legitimacy, enforcement capacity, power asymmetries

## Data Availability

No new data were created or analyzed in this study.
